# The use of direct oral challenge to confirm allergies to penicillin class antibiotics in Danish children

**DOI:** 10.1186/s12887-020-02407-z

**Published:** 2020-11-16

**Authors:** Thomas Krusenstjerna-Hafstrøm, Sune Rubak

**Affiliations:** 1 Department of Paediatric and Adolescents Medicine, Aarhus University Hospital, Palle Juul-Jensens Boulevard 99, 8200 Aarhus N, Denmark; 2 Danish Center of Pediatric Pulmonology and Allergology, Aarhus University Hospital, Palle Juul-Jensens Boulevard 99, 8200 Aarhus N, Denmark

## Abstract

**Background:**

A high number of children are referred to pediatric departments with a suspected allergic reaction to antibiotics. The prevalence of true allergy is considered to be significantly lower than shown from clinical history and symptoms alone. This study investigated the historical use of direct oral challenges at three specialist pediatric departments in Denmark.

**Methods:**

In this retrospective medical record review study, 141 children (69 boys and 72 girls) with a clinical history of suspected penicillin class allergy were investigated. A standardized questionnaire for drug allergy was completed in the beginning of the investigation, which also included a skin prick test (SPT), measurement of IgE to different types of penicillin, and a drug challenge (DC).

**Results:**

Only four (2.8%) of the patients referred for further investigation in our study had a positive DC. We found no correlation between a positive DC, positive SPT or elevated specific IgE. None of the patients with a positive DC reacted with a rash alone prior to investigation.

**Conclusions:**

Allergy to penicillin in children is rare and probably overestimated. In children reacting to penicillin with a rash alone, our study indicated that the rash was probably not related to allergy and treatment should thus be continued.

## Background

Only a small minority of children with suspected IgE-mediated allergy to penicillin is confirmed to be hypersensitive in direct oral challenge, which is the current reference standard. This study investigates the historical use of direct oral challenges at three specialist pediatric departments in Denmark. We speculated that most of the children referred with allergy to penicillin would show not to be.

Many children are referred to pediatric departments with a suspected allergic reaction to antibiotics. The prevalence of true allergy is considered to be significantly lower than indicated from the clinical history and symptoms alone. Among children, allergic reactions have been reported with an incidence as high as 10% of all prescriptions for antibiotics [1, 2]. However, drug-related side effects or disease-related symptoms are often mistaken for being an allergic reaction to antibiotics [3]. Examples of this are low risk symptoms, such as rashes or itching, which are rarely related to allergic reactions [4]. A large study from 2016 at the allergy clinic of the Montreal Children’s Hospital showed that among 818 patients suspected for amoxicillin allergy, only 5.8% tested positive in a graded drug challenge (DC) [5].

Penicillin is among the most commonly used antibiotics in children and thus often suspected to lead to allergic reactions in children [6]. These mistaken suspicions and often not validated diagnosis of allergy to penicillin increase the risk of doctors prescribing broad-spectrum antibiotics, which could potentially lead to a higher risk of antibiotic resistance. The American Academy of Allergy, Asthma and Immunology (AAAI) and European Network for Drug Allergy (ENDA)/ European Academy of Allergy and Clinical Immunology (EAACI) have recommend that investigation of suspected IgE-mediated penicillin allergy includes skin testing followed by an oral challenge with a therapeutic dose in skin test-negative individuals [7]. The usefulness of in vitro tests as commercial anti-penicillin IgE flurometric enzyme immunoassay is questionable [8]. This study investigated potential predictive symptoms and findings and discussed the diagnostics process in allergy to penicillin among children.

## Methods

This is a retrospective medical record review with no prospective interventional component. According to The Central Denmark Region Committee on Health Research Ethics, no formal ethics approval was required for this study. The study subjects were children followed in outpatient clinics at three Danish hospitals in Central Denmark Region, with a history of alleged allergy to a penicillin class antibiotic within the period from December 2007 to October 2011. In total, 141 children (69 boys and 72 girls) with an average age of 4.2 years (range, 0.33–15.3) were included. The local guideline was a graded two-step direct oral challenge/drug challenge (DC), with two total hours of observation to confirm antibiotic allergy or current tolerance, along with completion of a standardized ENDA drug allergy questionnaire. There was variable use of skin prick testing (SPT) (only in a subgroup (*n* = 52)), in vitro anti-penicillin and anti-cephalosporin specific IgE testing (*n* = 141), and measurements of total IgE level (104 individuals). The setup with SPT, DC and in vitro measurements of anti-penicillin and anti-cephalosporin specific IgE was in accordance with the European Network for Drug Allergy (ENDA) guidelines [9–11]. Specific IgE testing was done for penicillin G and V, amoxicillin, ampicillin, cefuroxime, ceftriaxone, cefamandole, cefotaxime, ceftazidime and penicillin minor determinant in all patients using ImmunoCAP Specific IgE 0–100 (Phadia APS, Allerød, Denmark) and a value > 0.35 kUA/L was considered positive. Total IgE was measured at the same time in 104 of the patients (74%) using ImmunoCAP Total IgE (Phadia APS, Allerød, Denmark). A standardized questionnaire (ENDA) for drug allergy was completed at the beginning of the investigation [12].

SPT was performed in 52 (37%) of the children, primarily at one of the study hospitals (98%). SPT with benzylpenicillin, ampicillin and amoxicillin (1.25 mg/ml) was performed together with SPT with the culprit drug according to the patient history; histamine 10 mg/ml (ALKAbello, Nordic, Hoersholm, Denmark) and isotonic sodium chloride were used as controls. SPT was used on the volar forearm and read after 20 min. If the increase of the largest diameter of the rash was > 3 mm, the test was considered positive.

The DC was performed as a graded oral challenge in all patients, including patients with positive SPT and/or positive specific IgE. Both a doctor and nurse were present all the time and the DC was performed following local anaphylaxis preparedness guidelines. The first dose was administered as one tenth of a normal treatment dose of penicillin/antibiotics (normal treatment dose: Amoxicillin, Penicillin V, Dicloxacillin and Amoxicillin + clavulanic acid = 16.7 mg/kg; Ampicillin = 25 mg/kg). If no reaction was seen, a second full dose was administered 60 min after the first dose. The patients were observed for 2 h before leaving the hospital. Afterwards, the parents observed the child at home for 7 days and reported by telephone if late onset symptoms requiring evaluation occurred. At another study hospital, Amoxicillin was used in the DC. If the DC was positive, a new test with the culprit drug was performed. At the other two study hospitals, the culprit drug was used from the beginning. In both scenarios the oral challenge was stopped if a clinical reaction occurred.

## Statistics

Hypothesis testing for continuous variables was done using the *t* test. Nominal statistical significance was set at a *p*-value < 0.05. All statistical analyses were performed using Sigmaplot 11.0, Systat Software.

## Results

### Symptoms and objective findings

Symptoms leading to referral of children to the pediatric departments are shown in Table 1.

**Table 1:** Distribution of symptoms

	Total	Negative	Positive
**Symptoms**	**137**	**133**	**4**
Urticaria	60 (44%)	58 (44%)	2 (50%)
Angioedema	13 (9.5%)	11 (8.3%)	2 (50%)
Itching rash			
small dotted macular exanthema	47 (34%)	47 (35%)	
large dotted macular exanthema	23 (17%)	23 (17%)	
confluent	30 (22%)	30 (23%)	
maculopapular exanthema	30 (22%)	30 (23%)	
Non itching rash			
small dotted macular exanthema	38 (28%)	37 (28%)	1 (25%)
large dotted macular exanthema	12 (8.8%)	12 (9.0%)	
confluent	19 (14%)	19 (14%)	
maculopapular exanthema	18 (13%)	17 (13%)	1 (25%)
Blisters	3 (2.2%)	3 (2.3%)	
Exfoliant			
< 5% body	6 (4.4%)	6 (4.5%)	
Conjunctivitis	3 (2.2%)	3 (2.3%)	
Dizziness	1 (0.7%)	1 (0.8%)	
Tachycardia	1 (0.7%)	1 (0.8%)	
Asthma	1 (0.7%)	1 (0.8%)	
Wheezing/Bronchospasm	4 (2.9%)	4 (3.0%)	
Lump in the throat	3 (2.2%)	3 (2.3%)	
Dyspnea	1 (0.7%)	1 (0.8%)	
Cough	4 (2.9%)	4 (3.0%)	

Total: all children; Negative: children with a negative provocation test; Positive: children with a positive provocation test

Most of the patients had several symptoms; the most common symptom was urticaria (44%; 95% confidence interval [CI], 35 to 52%) followed by a small dotted macular itching exanthema (34%; 95% CI, 26–42%) and a small dotted macular non-itching exanthema (28%; 95% CI, 20–35%).

The most common reason for starting treatment with antibiotics was sinusitis or otitis (57, 95% CI, 49–66%). Most children (91, 95% CI, 87–96%) had only reacted once to antibiotics before they were referred for a potential allergic reaction. Most often symptoms started within the first day of treatment (64, 95% CI, 56–72%) and persisted for less than 24 h after antibiotic treatment was stopped (48, 95% CI, 40–57%). Typically, no medication was given (77, 95% CI, 70–84%) to prevent the potential allergic reactions (Table 2).

**Table 2:** Description of reactions

	Total	Negative	Positive
**Reaction to AB before referral to DC**	**140**	**136**	**4**
Once	128 (91%)	124 (91%)	4 (100%)
More than once	12 (8.6%)	12 (8.8%)	
**Time period from AB dose to symptoms**	**135**	**131**	**4**
< 1 day	86 (64%)	84 (64%)	2 (50%)
1–2 days	27 (20%)	25 (19%)	2 (50%)
> 2 days	22 (16%)	22 (17%)	
**Duration of symptoms**	**137**	**133**	**4**
< 1 day	66 (48%)	63 (47%)	3 (75%)
> 1 day	60 (44%)	59 (44%)	1 (25%)
Unknown	11 (8.0%)	11 (8.3%)	
**Medical treatment**	**136**	**132**	**4**
None	105 (77%)	103 (78%)	2 (50%)
Antihistamines	30 (22%)	28 (21%)	2 (50%)
Corticosteroids	8 (5.8%)	8 (6.1%)	
Epinephrine	1 (0.7%)	1 (0.8%)	
Bronchodilators	2 (1.5%)	2 (1.5%)	
**Indication of AB treatment**	**133**	**129**	**4**
Airway infection	17 (13%)	16 (12%)	1 (25%)
Tonsillitis	21 (16%)	21 (16%)	
Sinusitis/otitis	76 (57%)	73 (57%)	3 (75%)
Other (impetigo, arthritis etc.)	15 (11%)	15 (12%)	
Unknown	4 (3.0%)	4 (3.1%)	

Total: all children; Negative: children with a negative provocation test; Positive: children with a positive provocation test

In all, 144 drug challenges were performed in the 141 patients and most were performed using amoxicillin (58, 95% CI, 50–67%) followed by penicillin V or G (36, 95% CI, 28–44% (Table 3).

**Table 3:** Antibiotics used for drug challenge

	Total	Negative	Positive
**Number of drug challenges**	**144**	**140 (97%)**	**4 (3%)**
Amoxicillin	84 (58%)	81 (58%)	3 (75%)
Penicillin V or G	52 (36%)	51 (36%)	1 (25%)
Dicloxacillin	5 (3%)	5 (4%)	
Ampicillin	2 (1%)	2 (1%)	
Amoxicillin+clavulanic acid	1 (1%)	1 (1%)	

Total: all children; Negative: children with a negative provocation test; Positive: children with a positive provocation test

### Diagnostic test

Positive DC was seen in four patients (2.8, 95% CI, 0.1–5.5%) Positive SPT was seen in one patient (1.9, 95% CI, − 1.9 - 5.8%) and one patient (0.7, 95% CI, − 0.7 - 2%) had a positive specific IgE.

Total IgE > 100 kU IgE/L was seen in 16% (95% CI, 9.1–24%) of which one child had a positive DC. Furthermore, 32% (95% CI, 23–41%) had a total IgE between 25 and 100 kU IgE/L (of which two children had a positive DC), and 52% (95% CI, 42–62%) had a total IgE < 25 kU IgE/L (of which one child had a positive DC).

### Characteristics of patients with a positive DC

Four patients (one boy and three girls aged 1.3 to 9.3 years) had a positive DC (Table 4).

**Table 4:** Characteristics of patients with a positive DC

Sex	Age	Symptoms prior to DC	SPT	Specific IgE*	Test drug	Dose	Symptoms	Time to symptoms
F	2.3	Small dotted macular exanthema Maculopapular exanthema Severe stomach pain	Neg	64	Amoxicillin	Full	Urticaria	30 min
M	9.3	Urticaria	–	262	Amoxicillin	Full	Urticaria	6 h
F	1.3	Angioedema	–	3	Penicillin	1/10	Angioedema	20 min
F	1.8	Urticaria Angioedema	–	57	Amoxicillin	1/10	Urticaria	30 min

*kU IgE/L

Three reacted to amoxicillin and one to penicillin V; all four patients had a negative specific IgE and one had a negative SP (the three others were not tested).

Of the four, one patient was referred with urticaria, one with angioedema, one with a combination of uritcaria and angioedema and the last one with a non-itching small dotted maculopapular exanthema and severe stomach pain (Tables 1 and 4).

All four were referred after their first allergic reaction; two had symptoms within the first day of antibiotic treatment and the other two within the first 2 days. Most of them (75%) had symptoms for less than 24 h. Two were not treated with any anti-allergic medication, and two were treated with antihistamine (Table 2).

During the DC, three patients developed urticaria, two 30 min after a dose (1/10 or full) and one six hours after a full dose. The last patient developed angioedema 20 min after 1/10 of a dose (Table 4).

### Characteristics of patients with a positive SPT

One patient had a positive SPT to amoxicillin, but DC and specific IgE were negative. Total IgE was 8 kU IgE/L. In this patient, the index reaction consisted of urticaria, together with an itching small dotted macular exanthema and wheezing. The symptoms started within the first treatment day and lasted up to 48 h, although the patient was treated with oral steroids and inhalations with a β_2_-agonist.

### Characteristics of patients with a positive specific IgE

One patient had a positive IgE (IgE = 0.45 kUA/L, penicillin minor determinant) but negative DC as well as negative SPT to penicillin; the total IgE was 1122 kU IgE/L. The patient developed a non-itching small dotted macular exanthema within the first day of treatment. The symptoms lasted for less than 24 h and no treatment was needed.

## Discussion

### Main findings

Only 2.8% of the patients referred to investigation of a potential allergic reaction to penicillin had clinically significant challenge reactions. This could indicate that allergy to penicillin in children has previously been overestimated as suggested by Esposito S et al. [13] We extended the guidelines recommended by EAACI Drug Allergy Interest Group [6] by performing DC in patients with a positive SPT or specific IgE, patients who would otherwise have been regarded as allergic to penicillin. This could partly explain the lower percentage in our study.

The study increased focus on which symptoms predict a true allergic reaction to antibiotics. A macular exanthema rash alone, whether itching or not, was not likely to be associated with a true allergy to antibiotics and antibiotic treatment should thus not be ended or changed. This corresponds to the division of symptoms into low-risk or high-risk as proposed by Vyles D et al. [4]

We found no correlation between a positive DC, positive SPT or high specific IgE. In non-IgE allergic reactions the SPT as well as specific IgE has shown a low sensitivity [14], and in immediate IgE-mediated allergic reactions, the concentration of IgE penicillin decreases if the time span is too long between the reaction and the SPT or measurement of IgE. The optimal timespan between reaction and test is between 1 and 12 months [15]. Therefore, it has been proposed to perform DC without SPT and IgE [16, 17], and only do a complete allergy work up (SPT, IgE with/without DC) in patients with a history suggesting anaphylaxis. This setup has been considered effective and safe to disregard hypersensitivity to penicillin. Although the same setup has been suggested for children [18, 19], the most recent Danish national guideline still recommends measuring specific IgE in all children before DC [20]. As concluded by Macy E et al. [8], our study did not find any evidence of specific IgE being a useful tool in diagnosing penicillin allergy.

### Limitations and strengths

One of the strengths of the study is that all children in the same geographical region during a period of about 5 years were included, thus being representative for a Danish population of children referred for a potential allergic reaction to antibiotics. Another strength is that disregarding symptoms, results of IgE or SPT, all patients underwent a DC, which showed no correlations between a positive DC, positive SPT or elevated specific IgE.

SPT was performed only in 37% of the children thus reducing the possibility to find a correlation with DC and specific IgE. We also used the culprit drug according to patient history; histamine and isotonic sodium chloride were used as controls. This could have reduced the sensitivity, as it would have been more correct to use the major determinant penicilloyl polylysine (Pre-Pen) or the minor determinants penicilloate and penilloate. However, Pre-pen was off the market from September 2004 to November 2009.

The study have limitations due to the different setup strategies for investigating allergy to antibiotics, and thus not using the same algorithm for validation of the diagnosis of allergy to antibiotics including SPT, IgE measurement and DC. We know that allergic reactions are divided into immediate (IgE mediated reactions) and non-immediate (T-cell mediated) reactions [14]. The oral DC used in this study consisted of two doses of antibiotics followed by a two-hour observation, thus primarily focused on the IgE-mediated allergic reaction and less on the detection of T-cell mediated reactions. This may explain why none of the symptomatic children had a positive DC after 2 days of antibiotic treatment (Table 2). In adults, using a seven-day DC detected 20% more patients with a positive diagnosis of allergy to antibiotics, including both IgE-mediated reactions and non-IgE /T-cell mediated allergic reactions [21, 22]. In children, a three-day DC with increasing doses of antibiotics detected 10% more children with a positive diagnosis of allergy to antibiotics with reactions showing between 24 and 48 hours [23]. Recently, it has been suggested that a five-day DC could increase the sensitivity of the diagnosis of non-IgE β-lactam allergy [24].

### Future research and clinical implications

Further studies to investigate the use of especially specific IgE for penicillin are needed. Al though we found no evidence of a correlation between DC and IgE, we speculate that IgE will be helpful in reactions suggesting anaphylaxis, where a DC could be contraindicated.

## Conclusion

We concluded that allergy to penicillin in children is rare and probably overestimated. Only 2.8% of the patients referred due to suspected allergy in our study had a positive DC.

In children reacting to antibiotics with a simple rash (itching or not) alone, our study indicates that the rash is most likely not related to allergy; antibiotic treatment should thus be continued.

In diagnosing allergy, measuring specific IgE provided no useful information in our study. We suggest that all patients should undergo a three- to seven-day DC to increase the sensitivity of the diagnosis of non-IgE β-lactam allergy.

## Data Availability

The dataset analysed in the current study are available from the corresponding author on reasonable request.

## Abbreviations

DC: Drug challenge
ENDA: European Network for Drug Allergy
SPT: Skin prick test
AAAI: American Academy of Allergy, Asthma and Immunology
EAACI: European Academy of Allergy and Clinical Immunology
